# Polymorphism of the DNA Base Excision Repair Genes in Keratoconus

**DOI:** 10.3390/ijms151119682

**Published:** 2014-10-29

**Authors:** Katarzyna A. Wojcik, Ewelina Synowiec, Katarzyna Sobierajczyk, Justyna Izdebska, Janusz Blasiak, Jerzy Szaflik, Jacek P. Szaflik

**Affiliations:** 1 Department of Molecular Genetics, University of Lodz, Pomorska 141/143, 90-236 Lodz, Poland; E-Mails: kwojcik@biol.uni.lodz.pl (K.A.W.); ewelinas@biol.uni.lodz.pl (E.S.); katarzyna2781@wp.pl (K.S.); jblasiak@biol.uni.lodz.pl (J.B.); 2 Department of Ophthalmology, Medical University of Warsaw, SPKSO Ophthalmic Hospital, Sierakowskiego 13, 03-709 Warsaw, Poland; E-Mails: justyna_izdebska@yahoo.es (J.I.); szaflik@szaflik.pl (J.S.)

**Keywords:** keratoconus, base excision repair, *NEIL1*, *PARP-1*, *POLG*, *XRCC1*

## Abstract

Keratoconus (KC) is a degenerative corneal disorder for which the exact pathogenesis is not yet known. Oxidative stress is reported to be associated with this disease. The stress may damage corneal biomolecules, including DNA, and such damage is primarily removed by base excision repair (BER). Variation in genes encoding BER components may influence the effectiveness of corneal cells to cope with oxidative stress. In the present work we genotyped 5 polymorphisms of 4 BER genes in 284 patients and 353 controls. The A/A genotype of the c.–1370T>A polymorphism of the DNA polymerase γ (*POLG*) gene was associated with increased occurrence of KC, while the A/T genotype was associated with decreased occurrence of KC. The A/G genotype and the A allele of the c.1196A>G polymorphism of the X-ray repair cross-complementing group 1 (*XRCC1*) were associated with increased, and the G/G genotype and the G allele, with decreased KC occurrence. Also, the C/T and T as well as C/C genotypes and alleles of the c.580C>T polymorphism of the same gene displayed relationship with KC occurrence. Neither the g.46438521G>C polymorphism of the Nei endonuclease VIII-like 1 (*NEIL1*) nor the c.2285T>C polymorphism of the poly(ADP-ribose) polymerase-1 (*PARP-1*) was associated with KC. In conclusion, the variability of the *XRCC1* and *POLG* genes may play a role in KC pathogenesis and determine the risk of this disease.

## 1. Introduction

Keratoconus (KC) is a progressive corneal disease that leads to worsening of visual quality. This disease usually appears in teenage years or early twenties and develops until the fourth decade of life [1]. KC occurs among all ethnicities, with incidence of approximately 1 per 2000. It is characterized by thinning of the cornea, resulting in its protrusion, a clinical hallmark of this disease. Changes in the corneal curvature may lead to myopia and irregular astigmatism [2]. Other signs include breakages in Bowman’s layer and deposition of iron in the basal layers of the corneal epithelium [1,3]. Features may also include fine parallel lines in the posterior stroma (Vogt’s striae), epithelial nebulae, anterior stromal scars and an increased visibility of corneal nerves. KC is typically a bilateral disease, although in the vast majority of cases it progresses asymmetrically [4,5].

Although KC is primarily an isolated condition, it may also coexist with several rare genetic disorders, including Down syndrome and Leber’s congenital amaurosis (as well as Ehlers–Danlos syndrome subtype VI, osteogenesis imperfecta and joint hypermobility) [6,7,8,9]. In addition, coexistence of hard contact lens wearing, eye rubbing, atopy of the eye and mechanical trauma with KC is well documented [10].

Despite intensive research, the exact cause of KC is not completely known. A significantly higher prevalence of KC in first degree relatives as well as high concordance in monozygotic twins indicate a genetic basis for KC [11,12,13]. From 6% to 23% of patients with KC exhibit a family history with autosomal dominant or recessive pattern of inheritance [1,14]. To date, multiple candidate genes were suggested as associated with KC [15]. Moreover, environmental factors seem to be implicated in progression of the disease [10].

Although pathogenesis of KC is not precisely determined, oxidative stress was reported to be associated with it [16,17,18]. The exposure of the cornea to endogenous and exogenous reactive oxygen species (ROS) can result in various types of molecular damages, affecting proteins, DNA and lipids. Alterations in DNA structure, if not repaired, can lead to genetic instability and mutations. Oxidative damage is implicated in a variety of eye diseases, including age-related macular degeneration (AMD), glaucoma, cataract and uveitis [19,20]. To protect genetic integrity, cells evolved several DNA repair pathways that eliminate many DNA damages [21]. Base excision repair (BER) is a primary repair mechanism of compact DNA lesions such as oxidized bases, abasic (AP) sites and can contribute to DNA single-strand break repair (SSBR) [22]. BER is initiated by a DNA glycosylase that recognizes base modification.

Nei endonuclease VIII-like 1 (NEIL1) is a bi-functional DNA glycosylase, involved in removing oxidative DNA lesions during BER [23]. NEIL1 recognizes oxidized pyrimidines, such as thymine glycol (Tg), 5-hydroxycytosine, 5-hydroxyuracil, 2,6-diamino-4-hydroxy-5-formamidopyrimidine (FapyG), 4,6-diamino-5-formamidopyrimidine (FapyA), and 8-hydroxyguanine [24,25]. This glycosylase cleaves damaged bases via βδ-elimination, generating 3'-phosphate and 5'-phosphate termini [23]. The NEIL1 DNA glycosylase was newly discovered as a mammalian ortholog of *E. coli* Nei enzyme and relatively little is known about the role of its genetic variability in physiology and pathology, which is mostly limited to cancer [26,27,28,29,30,31,32].

Poly(ADP-ribose) polymerase-1 (PARP-1) is involved in the regulation of several processes, including DNA repair, transcription, apoptosis, and inflammatory response [33,34]. PARP-1 is responsible for recognition of DNA strand breaks and polymerization of ADP-ribose from nicotinamide adenine dinucleotide (NAD^+^) in SSBR. This stimulates the recruitment of DNA repair proteins, including the X-ray repair cross-complementing group 1 (XRCC1) protein [35,36]. PARP-1 is a multifunction protein, which is involved not only in BER, but also in other DNA repair pathways and several non-repair processes, so it is an important component of cellular reaction to DNA damage, which may contribute to pathogenesis of many diseases, including cancer, cardiovascular diseases, diabetes, stroke and Alzheimer’s disease [37,38,39,40].

DNA polymerase γ, encoded by the polymerase gamma (*POLG*) gene, is the only DNA polymerase present in mammalian mitochondria, therefore it catalyses all mitochondrial DNA synthesis, also that involved in DNA repair, including short-patch BER [41,42]. In this pathway polymerase γ inserts a nucleotide into the gap to produce a substrate for DNA ligase. Several mitochondrial diseases are attributed to alterations in *POLG* [41,43]. Mutations in the *POLG* gene were reported to associate with progressive external ophthalmoplegia, a slowly progressing eye disease [42,44].

XRCC1 is another BER component, which acts as a scaffold protein in repair of base modifications and single strand breaks [36]. XRCC1 has a domain that acts as a protein-protein interface, whereby interacts with and coordinates the activity of the other BER proteins, including DNA ligase III, and DNA polymerase β [45,46,47,48]. Therefore XRCC1 participates in each step of repair of DNA damage in BER. Polymorphisms of *XRCC1* were reported to associate with eye diseases that were dependent upon the genetics and environmental factors. Results of meta-analysis showed an association between polymorphisms in *XRCC1* and increased risk of age-related cataract [49]. Another study found that the polymorphism in XRCC1 may also be associated with the progression of primary open-angle glaucoma [50].

In the present work we studied three BER genes, which are representative of the three main stages of this DNA repair pathway: base damage recognition (*PARP-1*), base removal (*NEIL1*) and repair synthesis (*POLG*). In addition, we studied the gene of an auxiliary BER factor, *XRCC1*. We aimed to explore the association of 5 single nucleotide polymorphisms (SNPs): the g.46438521G>C (rs4462560) polymorphism of the *NEIL1* gene, the c.2285T>C (rs1136410) polymorphism of the *PARP-1* gene, the c.–1370T>A (rs1054875) polymorphism of the *POLG* gene, the c.580C>T (rs1799782) and c.1196A>G (rs25487) polymorphisms of the *XRCC1* gene with KC occurrence. We also examined the association of some demographic and environmental risk factors with KC occurrence.

## 2. Results

### 2.1. Characteristics of the Study Population

Two hundred eighty four KC patients and 353 controls were enrolled in this study. Demographic variables and potential risk factors for KC of patients and controls are presented in Table 1. The mean age for KC patients were 36 ± 12.1 (range 14–68) and 63 ± 18.9 (range 19–100) for controls. There were significantly more subjects with positive family history for KC (the first degree relatives) among the patients in comparison to controls (12% *vs.* 2%, *p* < 0.001). We showed a significant difference between distribution of family history for KC (positive *vs.* negative), co-occurrence of visual impairment (yes *vs.* no) and distribution of allergies (yes *vs.* never) among KC patients and controls. These variables were further adjusted in multivariate logistic regression model for possible confounding factors of the main effect of the polymorphisms.

**Table 1:** Risk of keratoconus (KC) associated with age, sex, tobacco smoking, co-occurrence of visual disturbances, body mass index (BMI), allergies and family history of KC.

Feature	Controls (*n* = 353)		KC (*n* = 284)		*p*	OR (95% CI)	*p*_OR_
	Number	Frequency	Number	Frequency			
**Sex**					**<0.001**		
females	223	0.63	85	0.30		**0.24 (0.18–0.35)**	**<0.001**
males	130	0.37	199	0.70		**4.03 (2.88–5.62)**	**<0.001**
**Age**					**<0.001**	**0.92 (0.91–0.93)**	**<0.001**
Mean ± SD	63 ± 18.9		36 ± 12.1				
Range	19–100		14–68				
**Smoking**					0.746		
yes (current/former)	116	0.33	89	0.31		0.94 (0.67–1.32)	0.737
never	237	0.67	195	0.69		1.06 (0.76–1.48)	0.737
**KC in family**					**<0.001**		
yes	8	0.02	33	0.12		**5.61 (2.55–12.35)**	**<0.001**
no	345	0.98	251	0.88		**0.17 (0.08–0.39)**	**<0.001**
**BMI**					0.578		
≤25	149	0.42	131	0.46		1.16 (0.85–1.60)	0.347
25–30	119	0.34	92	0.32		0.94 (0.67–1.32)	0.729
≥30	85	0.24	61	0.21		0.87 (0.60–1.27)	0.470
**Visual impairment**					**<0.001**		
yes	114	0.32	197	0.69		**4.72 (3.36–6.62)**	**<0.001**
no	239	0.68	87	0.31		**0.21 (0.15–0.30)**	**<0.001**
**Allergies**					**<0.001**		
yes	44	0.12	80	0.28		**2.77 (1.84–4.16)**	**<0.001**
no	309	0.88	204	0.72		**0.36 (0.24–0.54)**	**<0.001**

*p*—values from χ^2^ tests; except *—values from *t*-test, comparing KC patients and controls; *p* < 0.05 are in bold; OR—odds ratio; 95% CI—95% confidence interval; *p*_OR_ values <0.05 along with corresponding ORs are in bold.

### 2.2. Relationship between Age, Sex, Tobacco Smoking, Co-Occurrence of Visual Disturbances, Body Mass Index (BMI), Allergies and Keratoconus (KC) in Family and the Risk of KC Independently of Genotype

We investigated the relationships between age, sex, tobacco smoking, co-occurrence of KC in family, visual disturbances, allergies and body mass index BMI and the risk of KC independently of genotype. We collated KC patients with controls according to these parameters (Table 1). We found that male sex, KC in family, co-occurrence of visual disturbances and allergies significantly increased the occurrence of KC, whereas age decreased this occurrence.

### 2.3. Polymorphisms of the Nei endonuclease VIII-like 1 (NEIL1), Poly(ADP-ribose) polymerase-1 (PARP-1), DNA Polymerase γ (POLG) and X-ray Repair Cross-Complementing Group 1 (XRCC1)Genes and KC Occurrence

The genotype and allele distributions of all studied polymorphisms in KC patients and controls are presented in Table 2. The observed genotypes frequencies for the c.580C>T SNP did not differ significantly from Hardy–Weinberg equilibrium (*p* > 0.05, data not shown) for KC subjects and controls. In our study we did not find any correlation between genotypes/alleles of the g.46438521G>C of *NEIL1* and c.2285T>C of *PARP-1* and KC occurrence. However, we showed a significant (*p* < 0.05) difference in the frequency distributions of genotypes of the c.–1370T>A polymorphism between the cases and controls. The presence of the A/A genotype was associated with increased occurrence of KC, whereas the A/T genotype was associated with decreased occurrence. We did not detected any correlation between alleles of the c.–1370T>A polymorphism and KC occurrence. We also observed a significant (*p* < 0.05) difference in the frequency distributions of polymorphisms in the *XRCC1* gene between the cases and controls. The presence of the C allele of the c.580C>T polymorphism was associated with increased occurrence of KC, but the T allele decreased it. Moreover, we found that the G/G genotype and the G allele of the c.1196A>G were associated with a protective effect against KC occurrence, whereas the A/G genotype and the A allele increased KC occurrence.

**Table 2:** Distribution of genotypes and alleles of the g.46438521G>C—*NEIL1*, c.2285T>C—*PARP-1*, c.–1370T>A—*POLG*, c.580C>T—*XRCC1* and c.1196A>G—*XRCC1* polymorphisms and odds ratio (OR) with 95% confidence interval (95% CI) in patients with KC and controls.

Polymorphism Genotype/Allele	Controls (*n* = 353)				KC (*n* = 284)				Crude OR (95% CI)	*p*		Adjusted OR ^a^ (95% CI)		*p*	
	Number		Frequency		Number		Frequency								
g.46438521G>C—*NEIL1*															
C/C	98		0.28		77		0.27		0.97 (0.68–1.37)	0.855		0.65 (0.39–1.09)		0.104	
C/G	240		0.68		188		0.66		0.92 (0.66–1.29)	0.632		1.37 (0.84–2.24)		0.210	
G/G	15		0.04		19		0.07		1.61 (0.81–3.24)	0.177		1.42 (0.49–4.14)		0.518	
χ^2^ = 1.856; *p* = 0.3953															
C	436		0.62		342		0.60		0.89 (0.67–1.20)	0.463		0.69 (0.44–1.07)		0.099	
G	270		0.38		226		0.40		1.12 (0.83–1.50)	0.463		1.45 (0.93–2.25)		0.099	
c.2285T>C—*PARP-1*															
A/A	239		0.68		191		0.67		0.98 (0.70–1.37)	0.904		0.95 (0.59–1.53)		0.825	
A/G	114		0.32		93		0.33		1.02 (0.73–1.42)	0.904		1.05 (0.65–1.70)		0.825	
G/G	0		0		0		0		–	–		–		–	
χ^2^ = 0.001; *p* = 0.9713															
A	592		0.84		475		0.84		0.98 (0.70–1.37)	0.904		0.95 (0.59–1.53)		0.825	
G	114		0.16		93		0.16		1.02 (0.73–1.42)	0.904		1.05 (0.65–1.70)		0.825	
c.–1370T>A—*POLG*															
A/A	46		0.13		62		0.22		1.86 (1.23–2.83)	0.004		2.71 (1.44–5.08)		0.002	
A/T	203		0.57		139		0.49		0.71 (0.52–0.97)	0.031		0.35 (0.22–0.56)		0.002	
T/T	104		0.30		83		0.29		0.99 (0.70–1.39)	0.948		1.27 (0.77–1.58)		0.358	
χ^2^ = 9.341; *p* = 0.0094															
A	295		0.42		263		0.46		1.22 (0.97–1.55)	0.091		1.22 (0.87–1.72)		0.250	
T	411		0.58		305		0.54		0.82 (0.65–1.03)	0.091		0.82 (0.58–1.15)		0.250	

^a^ OR adjusted for sex, age, co-occurrence of visual impairment, allergies, and family history for KC.

### 2.4. Gene–Gene Interaction and KC Occurrence

We also assessed the association between the occurrence of KC and combined genotypes of the g.46438521G>C—*NEIL1*, c.2285T>C—*PARP-1*, c.–1370T>A—*POLG*, c.580C>T—*XRCC1* and c.1196A>G—*XRCC1* polymorphisms. The distribution of such genotypes is shown in Supplementary Table S1, Supplementary Table S2, Supplementary Table S3, Supplementary Table S4, Supplementary Table S5, Supplementary Table S6, Supplementary Table S7, Supplementary Table S8 and Supplementary Table S9. We observed several associations between the occurrence of KC and combined genotypes. The presence of the C/C–A/A genotype of the g.46438521G>C—*NEIL1* and c.2285T>C—*PARP-1* polymorphisms was correlated with a decreased KC occurrence. The C/C–A/T genotype of the g.46438521G>C—*NEIL1* and c.–1370T>A—*POLG* polymorphisms was associated with increased KC occurrence, while C/G–A/A decreased this risk. The association between the C/C–A/G and the C/G–G/G genotypes of the g.46438521G>C—*NEIL1* and c.1196A>G—*XRCC1* polymorphisms and reduced KC occurrence were also found. On the other hand, the C/G–A/A genotype of the g.46438521G>C—*NEIL1* and c.1196A>G—*XRCC1* polymorphisms increased the occurrence of KC. Moreover, the A/A–A/A genotype of the c.2285T>C—*PARP-1* and c.–1370T>A—*POLG* polymorphisms was associated with increased KC occurrence, while the A/G-A/A genotype decreased this risk. The A/A–A/A genotype of the c.2285T>C—*PARP-1* and c.1196A>G—*XRCC1* polymorphisms was positively correlated with the occurrence of KC, whereas the A/A–G/G genotypes had a protective effect against KC. The A/T–C/T genotype of the c.–1370T>A—*POLG* and c.580C>T—*XRCC1* polymorphisms was associated with a significantly decreased risk of KC, while the A/A–C/C genotype of these polymorphisms increased this risk. Furthermore the occurrence of KC was positive correlated with the presence of the A/A–A/G genotype of the c.–1370T>A—*POLG* and c.1196A>G—*XRCC1* polymorphisms, while the A/T–G/G genotype demonstrated a protective effect.

### 2.5. Haplotypes and KC Occurrence

We also investigated the association between the occurrence of KC and haplotypes of the c.580C>T and c.1196A>G polymorphisms of the *XRCC1* gene. The distribution of such haplotypes is shown in Table 3. We found that the CA haplotype was correlated with increased KC occurrence, while the CG and TA haplotypes decreased it.

**Table 3:** Distribution of haplotypes of the c.580C>T and c.1196A>G polymorphisms of the *XRCC1* gene and OR with 95% CI in patients with KC and controls.

Haplotype	Controls (*n* = 353)		KC (*n* = 284)		OR (95% CI)	*p*
	Number	Frequency	Number	Frequency		
CA	675	0.48	635	0.56	**1.38 (1.83–1.62)**	**<0.001**
CG	641	0.45	457	0.40	**0.81 (0.69–0.95)**	**0.009**
TA	49	0.03	23	0.02	**0.57 (0.35–0.95)**	**0.030**
TG	41	0.03	21	0.02	0.63 (0.37–1.07)	0.089

*p* values <0.05 along with corresponding ORs are in bold.

### 2.6. Analysis of Polymorphic Variants of the NEIL1, PARP-1, POLG and XRCC1 Genes in Female and Male Groups, and the Risk of KC

The distribution of genotypes and allele frequencies of the five studied polymorphisms in *NEIL1*, *PARP-1*, *POLG* *and* *XRCC1* genes and the values obtained by the analysis of odds ratio (OR) in groups of females and males are shown in Supplementary Table S10. In analysis for the c.–1370T>A polymorphism, the A/A genotype was associated with a significantly increased risk of KC in women, whereas the A/T genotype decreased it. In males, the occurrence of KC was correlated with the presence of the C/C genotype and the C allele, while the C/T genotype and the T allele demonstrated a protective effect. Moreover, the G/G genotype and the G allele of the c.1196A>G polymorphism were associated with a significantly reduced risk of KC in both female and male groups, while the A allele increased it. In females, A/A genotype of this polymorphism also increased the risk of the disease.

## 3. Discussion

The pathogenesis of KC is still largely unclear, but multiple genetic and environmental factors are implicated in the development and progression of this disease [10,18]. Several genetic regions were identified through linkage studies in families affected with KC, including 3p14–q13; 5q14–q21; 5q32–q33; and 5q21.2; 14q11.2; 15q22–q24; 13q32; 2p24; 16q22–q23; 9q34; and 20q12 [51,52,53,54,55,56,57,58]. Besides, multiple genes were proposed to be associated with KC. Visual System Homeobox 1 (*VSX1*) was first gene involved with KC development [59]. The *VSX1* encodes a transcription factor, particularly engaged in the development of cornea [60,61]. Although several studies detected a correlation between mutation in *VSX1* and KC, many other studies did not find any relevant mutation in KC patients, indicating this gene has a role only in a small number of KC cases [62,63,64,65]. The r.57c>u mutation in the microRNA gene *miR-184*, located in 15q22–q25 region, was also detected in a family with KC [66]. Therefore, it is presumed that the variability of regulatory RNAs may be associated with KC pathogenesis. Dedicator of cytokinesis 9 (*DOCK9*) in 13q32 was another considered gene [15,67]. DOCK9 protein participates in activation of the cell division control protein 42 homolog (CDC42). Correlations between mutations in the *DOCK9* gene and KC susceptibility were shown. Additionally, changes in the zinc finger E-box binding homeobox 1 (*ZEB1*) and transforming growth factor, beta-induced (*TGFBI*) were linked with KC [68,69].

Besides genetic factors, oxidative stress is reported to be associated with the KC occurrence. Levels of aldehyde dehydrogenase Class 3, superoxide dismutase and glutathione S-transferase enzymes, which are responsible for elimination of ROS, were significantly decreased in KC corneas compared to controls [18,70]. The different distribution of stress-related enzymes detected in KC corneas may lead to increased susceptibility of tissue to oxidative damage. Moreover, KC corneas exhibited increased levels of cytotoxic byproducts of the lipid peroxidation and nitric oxide pathways, such as malondialdehyde and nitrotyrosine [17,71]. KC corneas also had an increased number of smaller-sized bands, such as deletions and mutations representing mtDNA [72]. Also, cytochrome oxidase (complex IV) subunit 1 (CO-1) is an important subunit of oxidative phosphorylation that is encoded in mitochondria, and a decrease in CO-1 in areas of corneal thinning was also reported [72].

Results of several studies also exhibited a positive association between KC and eye rubbing, visual impairment and allergies [1,10,73]. In our study, we investigated the relationship between some environmental and lifestyle factors and KC occurrence independently of genotype. Our results are in general agreement with those obtained in others laboratories. We reported significant correlation between visual impairment, allergies and an increased risk of KC. We also showed strong correlation between positive KC family history and KC occurrence, confirming results obtained in other laboratories [10,11,74]. Nevertheless we did not find any association between BMI, smoking and KC. We also observed a significant difference in age distributions between patients and controls. Because KC appears at a relatively young age, we were almost sure that control individuals would not develop this disease. The chance of late KC occurrence could be greater in younger individuals than in our control group. The patients searched for advice at different KC stages, so it was difficult to assess whether their actual state at the moment of diagnosis resulted from the severity of disease or its advance. Therefore, we did not include the onset time and any measure of severity in our analysis, as they might be highly uncertain.

In this work we also estimated the frequency of five SNPs in genes involved with the BER pathway in a Polish population. Our results indicated that the occurrence of KC may be correlated with the c.–1370T>A polymorphism of the *POLG* gene. However stratification analysis of the individuals according to sex showed a significant association of this polymorphism in females and lack of association among males. We also showed that the c.580C>T polymorphism of *XRCC1* decreased KC occurrence in males. We did not observe this association in females in stratification analysis of the individuals according to sex. These results indicate that the c.–1370T>A and c.580C>T polymorphisms respectively in females and males may play an important role in the risk of KC.

The analysis of the c.1196A>G polymorphism of *XRCC1* showed significant correlations with KC risk, which was also reported in stratification analysis in both sexes. We also reported several associations between KC occurrence and gene–gene interaction, which suggested that coexistence of several changes in DNA repair genes may lead to KC development.

Detected associations in our work may suggest that DNA repair genes, in particular genes involved in the BER pathway, may be involved in the pathogenesis of KC. Polymorphisms may bring functional changes in DNA repair genes and increase levels of oxidative DNA damage consequently inducing ocular diseases. We chose the c.580C>T and the c.1196A>G because these polymorphisms may influence function of XRCC1. The c.580C>T polymorphism is a missense substitution in the region involved in coordination of protein interactions [75,76]. We assume that the presence of the C allele may alter XRCC1 function as a scaffold protein, disturbing DNA repair. Decrease in repair efficiency may cause increased susceptibility to oxidative DNA damage in oxidative stress conditions, and increased accumulation of oxidative modification in the cornea, contributing to KC development. However, the c.1196A>G polymorphism can affect the poly (ADP-ribose) polymerase binding domain, leading to alternation of the efficiency of the repair process [76]. Results of several studies showed an association of the A allele of this polymorphism with increased levels of DNA damage, but several other studies reported the opposite tendency [77,78]. We think that the A allele may influence the structure of XRCC1 leading to changes in the detection of the DNA damage and the activation of the BER pathway. The role of c.–1370T>A in *POLG* is not known and requires explanation. Due to the location of this polymorphism in the 5' region of gene, we suspect that it may influence transcription efficiency. The presence of the A/A genotype of c.–1370T>A may decrease the activity of polymerase γ resulting in disturbance in repair of oxidative mtDNA damage in KC corneas. In our work we did not detect any significant association of the g.46438521G>C, located at the *NEIL1* regulatory region, and the c.2285T>C, causing a decrease enzymatic activity of PARP-1, with KC. However, we cannot exclude that another variation in *NEIL1* and *PARP-1* may have influence on the risk of KC. To our knowledge this is the first study investigating the role of *NEIL1,* *PARP-1*, *POLG* and *XRCC1* genes in KC, therefore further studies, performed on a larger population, are needed to obtain ultimate conclusions on such associations or its lack thereof.

## 4. Experimental Section

### 4.1. Ethic Description

The present study included 284 patients affected by KC and 353 controls recruited among patients from central Poland at the Department of Ophthalmology, Medical University of Warsaw (Warsaw, Poland).

The diagnosis of KC was based on ophthalmic examination, including best-corrected visual acuity, intraocular pressure, slit lamp examination and fundus examination [1,79,80]. In addition, topographical and pachymetric parameters on corneal topography (TMS4, Tomey, Nagoya, Japan), Orbscan corneal topographical and pachymetrical maps (Orbscan IIz, Bausch & Lomb, Rochester, NY, USA) were used to examine anomalies typical for KC. All clinical signs and the map patterns allowed diagnosis of KC. None of the control subjects exhibited any clinical signs of KC and everyone had healthy corneal endothelium on *in vivo* confocal microscopy (IVCM) and normal corneal pachymetry and topography, as described previously.

The study was approved by the Bioethics Committee of the Medical University of Warsaw (code decision: 18/2011 approved on 15 February 2011). Five microliters of peripheral blood from all samples were collected in tubes containing 10 mM ethylenediaminetetraacetic acid (EDTA) and stored at −20 °C. After obtaining informed consent, each subject was personally interviewed for information on demographic data and potential risk factors for KC. The information on age, body mass index (BMI), allergy, co-occurrence of visual impairment (hyperopia, astigmatism, myopia), and lifestyle habits, including smoking, and family history among first degree relatives for KC was obtained from each subject. Smoking was categorized due to current, former or never smokers. In addition, medical history was obtained from all subjects and no one reported any genetic disease. Table 1 presents characteristics of patients and controls.

### 4.2. Selection of Single Nucleotide Polymorphisms (SNPs) and Primer Design

We selected five SNPs in BER genes using the public domain of the National Center for Biotechnology Information at http://www.ncbi.nlm.nih.gov/snp (Bethesda, MD, USA). All SNPs have minor allele frequency (MAF) >0.05 in Caucasians (submitter population ID: HapMap-CEU; http://www.ncbi.nlm.nih.gov/snp). Finally, the g.46438521G>C in the 3' near gene of *NEIL1*, c.2285T>C in the exon of *PARP1*, c.–1370T>A in the 5' near gene of *POLG*, c.580C>T and c.1196A>G in the exons of *XRCC1* SNPs were selected for genotyping in this study. Then the published nucleotide sequence in ENSEMBL database (http://www.ensembl.org/index.html, Cambridge, UK) and Primer3 software (http://frodo.wi.mit.edu/, Tartu, Estonia) were used for primers design. The specificities of the high-resolution melting curve analysis (HRM) primer pairs were analyzed using Primer-BLAST software (http://www.ncbi.nlm.nih.gov/tools/primer-blast/index.cgi, Bethesda, MD, USA). TaqMan probe for c.2285T>C SNP was taken from the collection of Life Technologies (Gaithersburg, MD, USA).

### 4.3. DNA Extraction

DNA was extracted from peripheral blood leukocytes using AxyPrep™ Blood Genomic DNA Miniprep Kit (Axygen Biosciences, Union City, CA, USA), according to the manufacturer’s protocol. After extraction, DNA purity and concentration were assessed by comparing the absorbance at 260 and 280 nm.

### 4.4. High-Resolution Melting Curve Analysis (HRM) Genotyping

Genotyping of the g.46438521G>C polymorphism was performed by high-resolution melting curve analysis (HRM) on a C1000™ Thermal Cycler with CFX96™ Real-Time PCR Detection System (Bio-Rad, Hercules, CA, USA). PCR reactions were performed in 20 μL volume with 25 ng of genomic DNA, 1× KAPA HRM FAST Master Mix (containing EvaGreen^®^ dye), supplemented with 2.5 mM MgCl_2_ (Kapa Biosystems, Woburn, MA, USA) and 0.25 μM of each primer (Sigma-Aldrich, St. Louis, MO, USA). DNA fragment was amplified using the following primers: 5'-GGGCTTCTCAACTCATGGTC-3' and 5'-ACAGGAGAGACTGGGGACCT-3'. The PCR conditions included an initial denaturation at 95 °C for 2min, followed by 40 cycles of 95 °C for 5s, 60.3 °C for 30s. After amplification, HRM analysis data were collected from 71 to 86 °C, with each step raised by 0.2 °C (Figure S1). Melting curves were analyzed with Bio-Rad Precision Melt Analysis™ software.

### 4.5. TaqMan Genotyping

The c.2285T>C in *PARP1* genotyping analysis was performed using the TaqMan^®^ SNP Genotyping Assay on the same thermal cycler as in HRM analysis. The TaqMan assay was conducted using unlabeled PCR primer pair and 2 allele-specific TaqMan^®^ probes with a FAM™ or VIC^®^ dye labeled on the 5'-end as the reporter dyes. We used C_11468118_10 assay (Life Technologies, Foster City, CA, USA), in which probes with FAM™ hybridized to the T allele, whereas probes with VIC^®^ were specific to the C allele. PCR reactions were carried out in a total volume of 20 μL. The reaction conditions were as follows: 10 min at 95 °C, then 40 cycles consisting of 15 s denaturation at 92 °C, 1 min annealing and extending at 60 °C. The VIC and FAM fluorescence levels of the PCR products were measured at 60 °C for 1 min. The final products were analyzed on a CFX Manager Software, based on the dye component fluorescent emission data depicted in the X-Y scatter-plot (Figure S2). Each 96-well plate contained 94 samples with the unknown genotype and 2 reaction mixtures containing the reagents without DNA (no-template control).

### 4.6. Restriction Fragment Length Polymorphism (RFLP) Genotyping

The genotypes of the c.580C>T, c.–1370T>A and c.1196A>G polymorphisms were established using the polymerase chain reaction-restriction fragment length polymorphism (PCR-RFLP) method. A fragment covering the polymorphic site was amplified in a final reaction volume of 10 µL containing 0.25 U HotStarTaq Plus DNA Polymerase (Qiagen, Venlo, The Netherlands), 0.25 µM each primer, 200 μM deoxynucleotide triphosphates (dNTPs), 1 μL of 10× PCR buffer and 25 ng DNA.

Fragment containing the c.–1370T>A polymorphic site were amplified using the following primer sequences: forward 5'-CCGGGGCTTCTCTCTACC-3' and reverse 5'-GACCAACCGAGATCACACAG-3'. PCR was performed under the following conditions: initial denaturation at 95 °C for 5 min, 40 cycles at 95 °C for 30 s, 66 °C for 30 s, 72 °C for 1 min and a final elongation step at 72 °C for 5 min. After amplification, the 224 bp PCR products, containing the polymorphic site, were digested with 2 U of *Hpy*188I restriction enzyme (New England Biolabs, Ipswich, UK) at 37 °C for 16 h. The A allele has one cleavage site and was digested to 121 and 103bp fragments, whereas the T allele has no cleavage site and produces 224bp fragment only (Figure S3).

The c.580C>T polymorphism was determined using primers: forward 5'-TGAAGGAGGAGGATGAGAGC-3' and reverse 5'-TCAGACCCAGGAATCTGAGC-3'. The amplification protocol was set as 95 °C for 5 min, followed by 40 cycles of 95 °C for 30 s, 64 °C for 30 s, 72 °C for 1 min, and a final elongation step at 72 °C for 5 min. Then, five microliters of PCR amplicons were digested with 2 U of *Pvu*II restriction endonuclease (New England Biolabs, Ipswich, UK) in a final volume of 15 μL for 16 h at 37°C. The digested product with the T/T genotype showed two bands of 120 and 56 bp, the homozygote C/C only one band of 176 bp, whereas the C/T genotype produced three bands of 176, 120 and 56 bp (Figure S4).

For the c.1196A>G polymorphism we applied the following primers: forward: 5'-GGTCCTCCTTCCCTCATCTG-3'; reverse: 5'-TGCATCTCTCCCTTGGTCTC-3'. PCR cycling conditions were as follows: initial denaturation at 95 °C for 5 min, 40 cycles at 95 °C for 30 s, 64.5 °C for 30 s, 72 °C for 1 min and a final elongation step at 72 °C for 5 min. After amplification, a 5 μL aliquot of each PCR product was digested with 2 U of *Hpy*II restriction enzyme (New England Biolabs, Ipswich, UK) for 16 h at 37 °C. Fragment length after *Hpy*II digestion was 459 bp for the A/A genotype, 459, 277 and 182 bp for the A/G genotype and 277, 182 bp for the G/G genotype (Figure S5).

All amplification reactions were carried out on the C1000 Thermal Cycler. Products were verified by 8% polyacrylamide gel electrophoresis. Samples were visualized using ethidium bromide (0.5 mg/mL) and viewed in UV light. Electrophoresis was carried out at 5 V/cm in TBE buffer. A GeneRuler™ 100 bp (Fermentas, Hanover, MD, USA) or M100-500 DNA Ladder (BLIRT S.A., Gdansk, Poland) were used as a molecular mass marker. For quality control, 10% of samples were randomly genotyped again and the results were 100% concordant.

### 4.7. Statistical Analysis

Statistical analyses were performed with the SigmaPlot software, version 11.0 (Systat Software, Inc., San Jose, CA, USA), according to previous report [81]. The chi-square (χ^2^) test was used to assess the differences in frequency distributions of demographic and potential risk factors between patients and controls. For each SNP, the Hardy–Weinberg equilibrium (HWE) was assessed using χ^2^ test to compare the observed and expected genotype frequencies. The significance of the differences between distributions of genotypes and alleles in KC patients and controls were also performed by the χ^2^ test. The association between case-control status and each polymorphism, measured by the odds ratio (OR) and its corresponding 95% confidence interval (CI), was estimated using an unconditional multiple logistic regression model, both with and without adjustment for age, sex, co-occurrence of visual disturbances, allergies, and family status of KC. The association between the combined genotypes of the *NEIL1*, *PARP-1*, *POLG* and *XRCC1* polymorphisms and risk of this disease was also evaluated in the same way as single SNPs. Haplotypes were assessed for each subject on the basis of known genotypes and the PHASE software (http://stephenslab.uchicago.edu/software.html, Chicago, IL, USA) was used. Genetic effects of inferred haplotypes were analyzed in the same way as SNPs. Unconditional logistic regression analyses were also performed to assess the association between genotypes and risk for KC after stratification of the individuals according to sex. All statistical analyses were performed using SigmaPlot version 11.0 for Windows (Systat Software, Inc., San Jose, CA, USA).

## 5. Conclusions

Our results suggest the potential role of the c.–1370T>A polymorphism of *POLG* and the c.580C>T and c.1196A>G polymorphisms of *XRCC1* in KC pathogenesis.

## Supplementary Materials

Supplementary materials can be found at http://www.mdpi.com/1422-0067/15/11/19682/s1.
